# A tale of two intensive care units (ICUs): Baseline *Staphylococcus aureus* colonization and mupirocin susceptibility in neonatal and pediatric patients requiring intensive care

**DOI:** 10.1017/ice.2022.96

**Published:** 2023-03

**Authors:** Harbir S. Arora, Humera Khan, Haider Ailumerab, Girija Natarajan, Kathleen Meert, Hussein Salimnia, Rudolph Valentini, Ronald Thomas, Lynn Semproch, Basim I. Asmar, Eric J. McGrath

**Affiliations:** 1 Wayne State University School of Medicine, Detroit, Michigan; 2 Children’s Hospital of Michigan, Detroit, Michigan; 3 Central Michigan University College of Medicine, Mount Pleasant, Michigan; 4 Detroit Medical Center University Laboratories, Detroit, Michigan

## Abstract

**Objective:**

To assess the incidence rate of *S. aureus* colonization at baseline along with the mupirocin susceptibility (or resistance) rate in patients in a neonatal intensive care unit (NICU) and a pediatric intensive care unit (PICU) in conjunction with the implementation of universal decolonization as the standard of care.

**Design:**

Prospective cohort study.

**Setting:**

Children’s Hospital of Michigan (CHM) inpatient intensive care units (ICUs).

**Participants:**

Newly admitted pediatric patients to the CHM NICU or PICU aged between 1 day and ≤21 years.

**Interventions:**

Baseline and follow-up *S. aureus* screening cultures were obtained before patients underwent universal decolonization with mupirocin 2% antibiotic ointment (intranasal and umbilical) and chlorhexidine baths as standard of care to reduce CLABSI rates.

**Results:**

Baseline *S. aureus* colonization rates of new admissions to the CHM NICU and PICU were high at 32% and 29%, respectively. Baseline mupirocin susceptibility to any *S. aureus* growth was 98.4%. All baseline culture isolates whether positive for MRSA or MSSA, with one exception, had minimum inhibitory concentrations (MICs) of ≤0.19 µg/mL. All follow-up study cultures after universal decolonization at 7 days or beyond with any *S. aureus* growth had mupirocin MICs of ≤0.125 µg/mL.

**Conclusions:**

Baseline *S. aureus* colonization rates of new admissions to the CHM ICUs were high as was baseline mupirocin susceptibility. Follow-up cultures, albeit limited in number, did not detect increasing mupirocin MICs over 1 year, despite broad mupirocin exposure due to the implementation of universal decolonization.

Staphylococcal infections have garnered continued concern in both the neonatal intensive care units (NICUs) and pediatric intensive care units (PICUs) across the United States and internationally. Central-line–associated bloodstream infections (CLABSIs) due to *Staphylococcus aureus* are frequently encountered in those settings in children, and these infections lead to significant morbidity, mortality, and increased costs.^1^ Colonization with *S. aureus* often precedes invasive infection, especially for neonates in the intensive care unit (ICU).^2–5^

The Children’s Hospital of Michigan (CHM) implemented universal decolonization of newly admitted pediatric NICU and PICU patients with mupirocin 2% antibiotic ointment application (intranasal/umbilical) and concurrent chlorhexidine gluconate (CHG) baths in an effort to reduce CLABSI rates in December 2018.^6–8^ The nares and umbilicus are 2 areas that may become colonized with *S. aureus*.^7^ The *S. aureus* colonizing these sites may be resistant to mupirocin at baseline or resistance may develop due to exposure. *Staphylococcus* mupirocin resistance rates vary broadly but may increase with universal decolonization.^9–12^ At our institution, mupirocin was not included in routine *S. aureus* susceptibility testing and susceptibility was unknown.

The primary objective of this study was to assess the incidence of *S. aureus* colonization at baseline and mupirocin susceptibility (or resistance) in our NICU and PICU patients in conjunction with implementation of universal decolonization as the standard of care. The secondary objective was to assess changes in mupirocin susceptibility (or resistance) rates in patients testing positive for *S. aureus* in clinical cultures or screening and surveillance cultures after undergoing the universal decolonization regimen. A third objective of the study was to assess the effect of mupirocin with CHG baths on *S. aureus* colonization at treated sites.

## Methods

### Study procedures

A prospective convenience sample of newly admitted pediatric patients to the CHM NICU or PICU were offered participation in baseline and follow-up *S. aureus* screening cultures if they were aged between 1 day and ≤21 years. The Detroit Medical Center Research Review and Wayne State University Institutional Review Board approved this study (no. 111418MP2E). Informed consent and assent were obtained, as appropriate for age. After IRB approval and training the NICU and PICU staff, enrollment began on April 3, 2019. Training consisted of the study team meeting separately with the PICU and NICU medical and nursing staff to train them on the study protocol and to describe how the study related to the universal decolonization clinical protocol that was ordered for all new admissions in the electronic medical record (EMR). Unit-specific nursing managers reviewed the study status of all new admissions to the NICU or PICU along with the universal decolonization EMR orders every morning at the nursing “huddle” to ensure that no patients were missed. Exclusion criteria were being physically present in the CHM NICU/PICU setting for >24 hours prior to this admission, age >21 years, and prior treatment with mupirocin or chlorhexidine decolonization in the previous 4 weeks. After consenting, a culture swab from both nares and umbilicus were obtained for baseline *S. aureus* colonization screening. Enrollment ended abruptly on March 12, 2020, due to the COVID-19 pandemic.

### Clinical procedures

According to the CHM universal decolonization protocol, all patients newly admitted to the NICU or PICU received intranasal mupirocin ointment and topical umbilical decolonization (only infants aged <30 days) twice daily for 5 days. CHG baths were administered to patients as follows: once daily with 2% CHG cloth wipes (Sage Products, Cary, IL) for all patients ≥48 weeks of postmenstrual age (1) at admission to NICU or PICU with or without a central line in place or (2) when admitted to any other non-ICU unit but with a central line in place. CHG baths were continued for the duration of the ICU stay. CHG baths were administered 3 times per week for patients with postmenstrual age ≥42 and <48 weeks gestational age who were admitted to the NICU or PICU (1) with a central line, (2) with known MRSA colonization, or (3) who had been admitted to any other non-ICU unit with either a central line or history of MRSA colonization. CHG baths were also administered to patients ≥ 48 weeks gestational age the night before and morning of scheduled procedures or central-line insertion. For patients postmenstrual age ≥42 and <48 weeks, CHG baths were administered just before procedures or central-line insertion. Postmenstrual age (in weeks) is defined as the sum of gestational age (in weeks) and the number of weeks elapsed since birth. A postmenstrual age of 48 weeks indicates that the infant is 2 months past the due date of delivery (at 40 weeks).

Patients with positive clinical staphylococcal screening or surveillance culture not already receiving CHG baths (ie, patients admitted to medical floors before transfer to ICU or not meeting gestational age criteria), were administered CHG baths once when the screening culture turned positive. Additionally, those of gestational age ≥36 weeks or if gestational age was ≥28 weeks and postnatal age ≤4 weeks were administered another CHG bath 48 hours later. Patients who transferred out of the NICU or PICU before the 5 days of mupirocin completed therapy on the floor or ward.

The universal decolonization protocol comprised an admission EMR order set that included mupirocin, CHG baths, and clinical follow-up cultures. It was completed upon NICU or PICU admission without an initial *Staphylococcus* screen culture unless the patient was enrolled in the study. Subsequently, to assess long-term colonization status, clinical staphylococcal screening/surveillance cultures were obtained on days 28, 42, and 56 if the patient remained in ICU. Patients testing positive for MRSA on days 28, 42, or 56 were placed in contact isolation (MRSA only, per CHM policy) until a repeated culture (28-day intervals) was negative or universal decolonization was repeated (maximum, 3 times). Patients testing negative on day 28, 42, or 56 had repeated surveillance cultures at 14-day intervals.

After ∼5 months of enrollment, due to low numbers of patients remaining in ICUs after 28 days, study methods were amended so patients remaining in the NICU or PICU at day 7 (±2 days) and day 14 (±2 days) from study enrollment date had repeated staphylococcal screening cultures obtained from nares and umbilicus.

### Statistical analysis

Descriptive statistics were used to determine the initial *S. aureus* colonization rate before universal decolonization compared to rates of colonization or recolonization after universal decolonization. The incidence of mupirocin susceptibility among *S. aureus* isolated from screening/surveillance cultures was compared between samples collected from nares and umbilicus. Incidence of mupirocin susceptibility was compared between screening or surveillance baseline cultures (prior to receipt of universal decolonization) and any subsequent recolonization. The positive culture site was analyzed for colonization rate in nares versus umbilicus. Two-by-two (2 × 2) comparisons of proportions between NICU and PICU groups were conducted using the nonparametric Fisher exact test.

### Microbiology laboratory procedure

Swab cultures (eSwab sterile liquid collection kit, Becton-Dickinson, Sparks, MD) were plated on blood agar plates in the microbiology laboratory and were incubated for growth up to 48 hours. For positive cultures, determination of methicillin-resistant *S. aureus* (MRSA) and methicillin-susceptible *S. aureus* (MSSA) status was performed using the BD PHOENIX Automated Microbiology System (Becton-Dickinson) by oxacillin susceptibility. The mupirocin minimum inhibitory concentration (MIC) was obtained using mupirocin E-test strips (0.064–1024 µg/mL; AB Biodisk, Solna, Sweden). The break point for mupirocin susceptibility was ≤1.0 µg/mL.^13^

## Results

The study team approached 229 potential patients for study enrollment; however, 15 parents or guardians refused participation (5 in NICU, 10 in PICU). Ultimately, 214 patients were enrolled from both PICU and NICU, of whom 119 (55.6%) were male.

### Neonatal intensive care unit (NICU)

In the NICU, 93 patients were enrolled, representing 11% of the 820 NICU annual admissions. Table 1 summarizes demographic and clinical characteristics. The baseline colonization incidence of NICU patients upon admission was 30 (32%) of 93 from nares, umbilicus, or both. Table 2 summarizes baseline NICU colonization results. All enrolled patients underwent universal decolonization with mupirocin and CHG baths. Table 3 shows results for all NICU follow-up cultures. Table 4 shows CLABSI rates for the NICU (and PICU) before and after the intervention. See Supplementary Table 1 (online) for a 2017–2021 list of CHM ICU CLABSI pathogens.

**Table 1.** Patient Demographic and Clinical Characteristics

Variable	NICU	PICU
Total enrolled	93	121
Sex, male, n/N (%)	49/93 (52.7)	70/121 (58)
Mean chronological age mean (SD) (range)	35 (±4) d (1–139 d)	3.5 (±4.18) y (4 d–17 y)
Mean gestational age mean (SD) (range)	36.5 (±3.87) weeks (24–41 weeks)	N/A
Length of stay, mean (SD) (range)	9.72 (±1.4) d (1–108 d)	6.26 (±14) d (1–115 d)

Note. NICU, neonatal intensive care unit; PICU, pediatric intensive care unit.

**Table 2.** Baseline NICU and PICU Colonization Results Based on Site Swabbed

Variable	NICU Culture Positive/Any Site Positive (%)	PICU Culture Positive/Any Site Positive (%)	*P* Value
Baseline *S. aureus* colonization	30/93 (32)	35/121 (29)	.654
**Nasal culture, baseline**	22/93 (24)	33/121 (27)	.636
Positive for MRSA Positive for MSSA	10/93 (11) 12/93 (13)	10/121 (8) 23/121 (19)	.637 .266
**Umbilical culture positive, baseline**	14/88 (16)^a^	6/119 (5)^a^	.009**
Positive for MRSA Positive for MSSA	2/88 (2) 12/88 (14)	1/119 (1) 5/119 (4)	.576 .020*
**Both sites positive, baseline** ^**b**^	6^c^	4^c^	.337
Both sites positive for MRSA^b^ Both sites positive for MSSA	2 MRSA 4 MSSA	4 MSSA	.612 NA

Note. NICU, neonatal intensive care unit; PICU, pediatric intensive care unit; MRSA, methicillin-resistant *Staphylococcus aureus*; MSSA, methicillin-susceptible *Staphylococcus aureus*; NA, not applicable.

a 5 NICU and 2 PICU patients did not have umbilical culture obtained at baseline.

b Numbers reflect raw numbers of cases with both culture sites positive.

c No NICU or PICU patients tested positive for both MSSA and MRSA at baseline.

* Statistically significant at P ≤ .05.

** Statistically significant at P ≤ .01.

**Table 3.** Follow-up Study Impact and Clinical Culture Results

Day From Enrollment and Site Swabbed	No. of Patients Cultured for Study or Clinical	No. of Patients Cultured With Positive Growth	Note
**NICU**			
Day 7, nose	16	0	
Day 7, umbilical	16	1	1 culture positive for MSSA and patient’s baseline umbilical culture grew MSSA
Day 14, nose	9	0	
Day 14, umbilical	9	0	
Day 28, nose	2	0	
Day 28, umbilical	2	0	
Day 42, umbilical^a^	1	1	1 culture positive for MSSA and patient’s baseline nasal culture grew MRSA
Day 56, nose	1	1	1 culture positive for MSSA and patient’s baseline nasal and umbilical cultures grew MSSA
Day 56, umbilical	1	0	
**PICU**			
Day 7, nose	9	1	1 culture positive for MRSA
Day 7, umbilical	9	0	
Day 14, nose	3	1	1 culture positive for MSSA
Day 14, umbilical	3	0	
Day 28, nose	4	0	
Day 28, umbilical	3	0	
Day 42, nose	1	1	1 positive for MSSA and patient’s baseline nasal and umbilical cultures grew MSSA; cultures were clear at day 7 but nares culture grew MSSA at day 14 and bothe cultures were negative at day 28 as well
Day 42, umbilical^a^	1	0	

Note. NICU, neonatal intensive care unit; PICU, pediatric intensive care unit; MSSA, methicillin-susceptible *Staphylococcus aureus*; MRSA, methicillin-resistant *Staphylococcus aureus*.

a Inadvertently, no clinical cultures were obtained on day 42 in NICU (nose) and Day 56 in PICU (nose, umbilical) by clinical service.

**Table 4.** NICU and PICU CLABSI Rates per 1,000 Device Days Before and After the Intervention^a^

Year	NICU	PICU
2017	1.4	1.25
2018	1.49	1.51
2019	1.19	1.14
2020	0.74	0.86
2021	1.26	0.50

Note. NICU, neonatal intensive care unit; PICU, pediatric intensive care unit; CLABSI, central-line–associated bloodstream infection.

a Universal decolonization of all new admissions to the NICU and PICU was instituted at Children’s Hospital of Michigan in December, 2018.

#### Impact of cultures in the NICU—Nasal and umbilical on day 7

In total, 16 infants had follow-up day 7 nasal culture. Of these 16 infants, the 3 who were positive for MRSA at baseline from nares were negative on day 7, and all 13 (100%) of 13 who were negative at baseline remained negative on day 7. Intranasal mupirocin with CHG baths was a successful decolonization protocol in 3 (100%) of 3 patients. Additionally, the 13 patients who were negative at baseline all remained negative on day 7, confirming prevention of new nasal colonization. Furthermore, 16 infants had a follow-up umbilical culture on day 7. Of these 16 infants, the 1 infant positive for MRSA at baseline was negative on day 7. Of the 2 infants positive for MSSA at baseline, one infant remained positive on day 7 and the other infant was negative. All 13 (100%) of 13 infants negative at baseline remained negative on day 7. Thus, mupirocin with CHG baths achieved eradication of colonization at the umbilical site in 2 (67%) of 3 infants and effectively prevented new umbilical colonization for the rest.

#### Impact of cultures in the NICU—Nasal and umbilical on day 14

In total, 9 infants had follow-up nasal cultures on day 14. All 9 infants were negative on day 7 and remained negative on day 14. Thus, 9 (100%) of 9 infants remained negative for nasal colonization at 14 days. Also, 9 infants had a follow-up umbilical culture on day 14. Of these 9 infants, 1 infant who had been positive for MSSA on day 7 had negative growth on day 14. All 8 (100%) of 8 infants who tested negative on day 7 were still negative on day 14. The effectiveness of the umbilical mupirocin application with CHG baths increased from 50% at 7 days to 100% at 14 days. Additionally, no new case of umbilical colonization was identified at 14 days.

### Pediatric intensive care unit (PICU)

In the PICU, 121 patients were enrolled, which represented 6% of the 1,937 annual admissions. Table 1 summarizes the demographic and clinical characteristics of these patients. Baseline cultures upon PICU admission revealed culture growth in 35 (29%) of 121 samples from nares, umbilicus, or both. Table 2 summarizes baseline PICU colonization results.

None of the 10 patients who tested positive for MRSA in nasal cultures were positive for MRSA in umbilical cultures; however, 1 of 2 PICU patients did not have baseline umbilical culture obtained. Also, 4 patients tested positive for MSSA from both their nares and umbilicus at baseline. One patient with MRSA growth on umbilical culture was negative on baseline nasal culture. One patient with MSSA growth on umbilical culture was also negative on baseline nasal culture. All enrolled patients underwent decolonization with mupirocin and had CHG baths according to the clinical protocol. Table 3 shows results for follow-up cultures in the PICU and Table 4 lists the annual CLABSI rates.

#### Impact of cultures in the PICU—Nasal and umbilical on day 7

In total, 9 patients had follow-up nasal cultures on day 7. Of these 9 children, the 1 patient who was positive for MRSA at baseline in the nares culture remained positive on day 7. The 2 patients positive for MSSA on baseline culture were negative at day 7. All 6 (100%) of 6 patients with cultures that were negative at baseline remained negative on day 7. Intranasal mupirocin with CHG baths was effective in 2 (66%) of 3 PICU patients at 7 days, along with apparent prevention of the development of new nasal colonization in all remaining 6 patients. Moreover, 9 patients had a follow-up umbilical culture on day 7. Of these 9 children, the 1 patient who had had a positive MSSA culture at baseline was negative on day 7. Thus, mupirocin with CHG baths eradicated colonization of the single colonized case and prevented new colonization in the rest.

#### Impact of cultures in the PICU—Nasal and umbilical on day 14

In total, 3 patients had follow-up nasal cultures on day 14. Of these 3 children, the 1 patient positive for MRSA on day 7 was also negative on day 14. The growth of MSSA on day 14 was noted in 1 patient who had had a negative nasal culture on day 7; this patient had been positive for MSSA at baseline from both umbilicus and nares. Finally, 1 patient was negative on day 7 and remained negative at day 14. Therefore, intranasal mupirocin with CHG baths were effective in 1 (50%) of 2 patients and a third pateint remained uncolonized from baseline. Also, 3 patients in the PICU had follow-up umbilical cultures on day 14, and all 3 had negative cultures on day 7 and remained negative at day 14.

### Mupirocin susceptibility

Of 65 baseline cultures with growth of *S. aureus*, 64 (98.4%) were deemed mupirocin susceptible, with MICs ≤0.19 µg/mL. Only 1 culture of the 75 positive *S. aureus* screening cultures at baseline revealed MRSA identified from a baseline nasal culture of a NICU infant, with a mupirocin MIC of 1,024 µg/mL, suggesting high-level resistance. All other baseline positive culture isolates, whether MRSA or MSSA, had MICs ≤0.19 µg/mL. Additionally, all follow-up study cultures from 7 days after universal decolonization and beyond with any *S. aureus* growth had mupirocin MICs ≤0.125 µg/mL (Supplementary Fig. 1 online).

### Clinical disease

Routine surveillance by the CHM epidemiology team for *S. aureus* bacteremia in all patients in the CHM NICU and PICU were compared with enrolled study patients and no breakthrough infections were detected.

## Discussion

In our study, baseline *S. aureus* colonization rates in a sample of new admissions to the CHM NICU and PICU were high at 32% and 29%, respectively. Among *S. aureus* isolates from baseline culture, mupirocin susceptibility was 98.4%. A limited number of postdecolonization follow-up cultures did not detect increasing mupirocin MICs nor the development of resistance.

Colonization of nares and umbilicus with *S. aureus* (MRSA) in infants can cause significant mortality and morbidity as invasive infections.^3^ Colonized infants had a significantly higher rate of MRSA infection compared to those infants who were not colonized (26% vs 2%). Using genotyping analysis of 84 episodes of MRSA infection, previous colonization was detected in 68 episodes (81%), and the clinical and colonization isolates were indistinguishable in 63 episodes. Nares and umbilicus were the 2 most commonly colonized sites, 71% and 60%, respectively. Moreover, 69% of these infants were positive at first culture and up to 89% were positive at the first 2 cultures.^3^ In another large study of 783 infants in a NICU, 323 infants (>40%) were colonized with MRSA.^2^ A meta-analysis of 18 studies estimated a relative risk of 24.2 among colonized patients to develop a MRSA infection during hospitalization.^14^ Furthermore, rates of MRSA colonization were 1.9% on NICU or PICU admission. The rate of colonization among infants born outside reporting centers was significantly higher (5.8% vs 0.2%), indicating a community source. The pooled acquisition rate of MRSA colonization in pediatric patients was 4.1% (95% CI, 1.2%–8.6%) during their NICU or PICU stay and was 6.1% among NICU patients alone.^14^ Our findings indicate that a notable percentage of patients colonized with *S. aureus* upon ICU admission provide a potential reservoir of transmission and potential for increased hospital-acquired infections.

A national surveillance study reported invasive MRSA infections, with 4,872 cases of infections occurring among 4,445 persons (8% had >1 infection) in 2011. Among these infections, 2,912 (60%) were healthcare-associated community onset, 868 (18%) were hospital onset, and 966 (20%) were community associated.^15^ Although US rates of invasive MRSA infections among adults have decreased,^15–18^ these numbers are on the rise in the pediatric population.^19–21^ To be sure, MRSA and MSSA strains of *S. aureus* cause invasive infections responsible for significant morbidity and mortality and, in some studies, MSSA caused more infections and deaths in infants than MRSA.^22^ The burden of *S. aureus* infections among infants in NICUs is higher than is generally perceived.^23–26^ *S. aureus* was the second most common cause of late-onset sepsis among extremely premature neonates admitted in 12 US centers, causing 12% of infections.^24^

Mere identification of colonization, contact isolation, and placement in cohorts, have been unreliable and ineffective in decreasing colonization rates in neonates.^27^ The data on mupirocin decolonization in neonates resulting in decreased rates of clinical staphylococcal infections are sparse, but the decolonization data alone are strong, suggesting the effectiveness of mupirocin.^28–31^ After a failed attempt at targeted intranasal mupirocin among colonized neonates, universal use in previously admitted neonates and new admissions successfully controlled a MRSA outbreak.^29^

Targeted use of intranasal mupirocin among colonized neonates has been studied and compared with no intervention. An impressive colonization rate of 25% (130 infants) was noted in a study of 525 neonates^7^ with the rate of clinical infection due to MRSA being significantly higher in those colonized (10.2%) than in those not colonized (2.3%). Among colonized infants, 5 days of twice daily topical mupirocin application to nares and umbilicus yielded MRSA infection rates that were significantly lower (3.2%) compared to colonized infants without intervention (16%). All MRSA isolates in that study were mupirocin susceptible.^7^ Although the development of mupirocin resistance after prolonged use has been observed,^10,11^ the development of resistance after short courses is rarely reported unless an isolate with plasmid-mediated high-level resistance is detected.^30,32–34^ A low rate of mupirocin resistance was observed in our study.

This study had several limitations. These data reflect the experience at a single center. Because patients received both mupirocin and CHG baths, the individual impact of each intervention could not be separated. A low number of mupirocin impact and clinical follow-up cultures were conducted, which was addressed by a study protocol amendment. No weekend admissions (Friday or Saturday admissions) were screened for enrollment because study volunteers worked weekdays. The convenience sample captured 11% and 6% of NICU and PICU admissions, respectively, for which sampling bias cannot be excluded. Cultures on day 7 may reflect mere suppression of *S. aureus* growth (rather than true decolonization) because mupirocin use ended near day 7. Study patients who were colonized with *S. aureus* were later cleared of colonization after universal decolonization and later regrew *S. aureus*, which has been reported previously,^35,36^ but due to the limited number of patients with regrowth, no further conclusions were drawn. Long-term information on mupirocin susceptibility in the ICUs was not collected, and *S. aureus* susceptibility to CHG was not assessed.

In conclusion, baseline *S. aureus* colonization rates of new admissions to the CHM NICU and PICU were high at 32% and 29%, respectively. Baseline mupirocin susceptibility was also high at 98.4% among *S. aureus* isolates. Follow-up cultures, albeit limited, did not detect increasing mupirocin MICs or resistance over 1-year study duration, despite broad mupirocin and CHG exposures related to the implementation of a universal decolonization protocol for all new admissions to the CHM NICU and PICU.

## Supplementary material

For supplementary material accompanying this paper visit https://doi.org/10.1017/ice.2022.96. click here to view supplementary material
