# Supplementary material for: A tale of two intensive care units (ICUs): Baseline Staphylococcus aureus colonization and mupirocin susceptibility in neonatal and pediatric patients requiring intensive care
Source: Infect Control Hosp Epidemiol. 2022 Apr 22;44(3):447–52. doi: 10.1017/ice.2022.96 (PMC10015265; doi:10.1017/ice.2022.96)
Supplement: Supplementary file 1 [file S0899823X22000964sup.zip › S0899823X22000964sup001.docx]

| **NICU** | NICU Nasal | NICU Umbilical | NICU Nasal | NICU Umbilical | NICU Nasal | NICU Umbilical | NICU Nasal | NICU Umbilical | NICU Nasal | NICU Umbilical | NICU Nasal | NICU Umbilical |
| --- | --- | --- | --- | --- | --- | --- | --- | --- | --- | --- | --- | --- |
| **Mupirocin MIC (µg)** | **Baseline** | **Baseline** | **Day 7** | **Day 7** | **Day 14** | **Day 14** | **Day 28** | **Day 28** | **Day 42** | **Day 42** | **Day 56** | **Day 56** |
| ***0.064*** | 10  (MRSA, 4  MSSA, 6) | 3  (MSSA) |  |  |  |  |  |  |  | 1  (MSSA) |  |  |
| ***0.094*** | 8  (MRSA, 5  MSSA, 3) | 7  (MRSA, 1  MSSA, 6) |  |  |  |  |  |  |  |  | 1  (MSSA) |  |
| ***0.125*** | 3  (MSSA) | 3  (MSSA) |  | 1  (MSSA) |  |  |  |  |  |  |  |  |
| ***0.19*** |  | 1  (MRSA) |  |  |  |  |  |  |  |  |  |  |
| ***1024*** | 1  (MRSA) |  |  |  |  |  |  |  |  |  |  |  |
|  | | | | | | | | | | | | |
| **PICU** | PICU Nasal | PICU Umbilical | PICU Nasal | PICU Umbilical | PICU Nasal | PICU Umbilical | PICU Nasal | PICU Umbilical | PICU Nasal | PICU Umbilical | PICU Nasal | PICU Umbilical |
| **Mupirocin MIC (µg)** | **Baseline** | **Baseline** | **Day 7** | **Day 7** | **Day 14** | **Day 14** | **Day 28** | **Day 28** | **Day 42** | **Day 42** | **Day 56** | **Day 56** |
| ***0.064*** | 15  (MRSA, 5  MSSA, 10) | 5  (MRSA, 1  MSSA, 5) | 1  (MRSA) |  |  |  |  |  | 1  (MSSA) |  |  |  |
| ***0.094*** | 13  (MRSA, 4  MSSA, 9) | 1  (MSSA) |  |  |  |  |  |  |  |  |  |  |
| ***0.125*** | 3  (MRSA, 1  MSSA, 2) |  |  |  | 1  (MSSA) |  |  |  |  |  |  |  |
| ***0.19*** | 2  (MSSA) |  |  |  |  |  |  |  |  |  |  |  |

ABBREVIATIONS: NICU, neonatal intensive care unit; PICU, pediatric intensive care unit; MIC; minimum inhibitory concentration; MRSA, methicillin-resistant *Staphylococcus aureus*; MSSA, methicillin-susceptible *Staphylococcus aureus*
