# Supplementary material for: A tale of two intensive care units (ICUs): Baseline Staphylococcus aureus colonization and mupirocin susceptibility in neonatal and pediatric patients requiring intensive care
Source: Infect Control Hosp Epidemiol. 2022 Apr 22;44(3):447–52. doi: 10.1017/ice.2022.96 (PMC10015265; doi:10.1017/ice.2022.96)
Supplement: Supplementary file 1 [file S0899823X22000964sup.zip › S0899823X22000964sup002.docx]

List of CLABSI Pathogens from 5 years in CHM NICU and PICU

| **2017** | **NICU (9)** | **PICU (7)** |
| --- | --- | --- |
|  | *Staphylococcus epidermidis* | *Staphylococcus epidermidis* |
|  | MSSA | *Staphylococcus epidermidis* |
|  | MRSA | Group B Streptococcus (GBS) |
|  | *Enterococcus faecalis* | *Stenotrophomonas maltophilia* |
|  | *Enterococcus faecalis* | *Pseudomonas aeruginosa; Enterococcus faecium* |
|  | *Klebsiella pneumoniae* | *Klebsiella pneumoniae* |
|  | *Klebsiella oxytoca* | *Candida parapsilosis; Stenotrophomonas maltophilia; Chryseobacter (Flavo.) indologenes; Ochrobactrum anthropi* |
|  | *Klebsiella pneumonia, MSSA, Staphylococcus haemolyticus* |  |
|  | *Candida parapsilosis* |  |
|  |  |  |
| **2018** | **NICU (9)** | **PICU (9)** |
|  | *Staphylococcus epidermidis* | MRSA |
|  | MRSA | MRSA |
|  | Group B Streptococcus (GBS) | MRSA |
|  | *Serratia marcescens* | MSSA |
|  | *Pseudomonas aeruginosa* | *Enterococcus faecalis, Staphylococcus hominis* |
|  | *Enterococcus faecalis, Staphylococcus epidermidis* | *Klebsiella pneumoniae* |
|  | *Klebsiella pneumoniae, Pseudomnoas aeruginosa* | *Enterobacter cloacae* |
|  | *Pseudomonas aeruginosa, Stenotrophomonas maltophilia* | *Candida tropicalis* |
|  | *Candida parapsilosis, MRSA* | *Candida dublinensis* |
|  |  |  |
| **2019** | **NICU (7)** | **PICU (6)** |
|  | Staphylococcus epidermidis | *Klebsiella pneumoniae* |
|  | MSSA | *Klebsiella pneumoniae* |
|  | MSSA | *Klebsiella pneumoniae* |
|  | Enterococcus faecalis | *Enterobacter cloacae* |
|  | Pseudomonas aeruginosa | *Candida parapsilosis* |
|  | Escherichia coli | *Malasezia pachydermidis* |
|  | Candida albicans |  |
|  |  |  |
| **2020** | **NICU (4)** | **PICU (5)** |
|  | Staphylococcus epidermidis | MRSA |
|  | Staphylococcus epidermidis | MSSA |
|  | *Klebsiella pneumoniae* | *Hafnia alvei* |
|  | *Serratia marcescens* | *Enterococcus faecalis* |
|  |  | *Enterobacter sp.* |
|  |  |  |
| **2021** | **NICU (8)** | **PICU (3)** |
|  | Staphylococcus epidermidis | *Staphylococcus epidermidis* |
|  | *MRSA* | *Enterococcus faecalis* |
|  | *Enterobacter cloacae* | Escherichia coli |
|  | Escherichia coli |  |
|  | *Enterococcus faecalis, Serratia marcescens* |  |
|  | *Klebsiella pneumonia,* MSSA |  |
|  | *Candida tropicalis* |  |
|  | *Candida tropicalis* |  |

Abbreviations: CLABSI, Central Line Associated Bloodstream Infection; CHM, Children’s Hospital of Michigan; NICU, Neonatal Intensive Care Unit; PICU, Pediatric Intensive Care Unit; MRSA, methicillin-resistant *Staphylococcus aureus*; MSSA, methicillin-susceptible *Staphylococcus aureus*
